# Postural control depends on early visual experience

**DOI:** 10.1167/jov.24.9.3

**Published:** 2024-09-03

**Authors:** Kirsten Hötting, Idris Shareef, Ann-Kathrin Rogge, Daniel Hamacher, Astrid Zech, Ramesh Kekunnaya, Beula Christy, Brigitte Röder

**Affiliations:** 1 Biological Psychology and Neuropsychology, University of Hamburg, Hamburg, Germany; 2 Department of Nursing and Management, Hamburg University of Applied Sciences, Hamburg, Germany; 3 Jasti V Ramanamma Children's Eye Care Center, Child Sight Institute, LV Prasad Eye Institute, Hyderabad, India; 4 Department of Psychology, University of Nevada, Reno, NV, USA; 5 Max Planck School of Cognition, Max Planck Institute for Human Cognitive and Brain Sciences, Leipzig, Germany; 6 Institute of Sports Science, Friedrich Schiller University Jena, Jena, Germany; 7 Institute for Vision Rehabilitation, LV Prasad Eye Institute, Hyderabad, India

**Keywords:** blindness, sight recovery, locomotion, balance, sensitive periods

## Abstract

The present study investigated the role of early visual experience in the development of postural control (balance) and locomotion (gait). In a cross-sectional design, balance and gait were assessed in 59 participants (ages 7–43 years) with a history of (a) transient congenital blindness, (b) transient late-onset blindness, (c) permanent congenitally blindness, or (d) permanent late-onset blindness, as well as in normally sighted controls. Cataract-reversal participants who experienced a transient phase of blindness and gained sight through cataract removal surgery showed worse balance performance compared with sighted controls even when tested with eyes closed. Individuals with reversed congenital cataracts performed worse than individuals with reversed developmental (late emerging) cataracts. Balance performance in congenitally cataract-reversal participants when tested with eyes closed was not significantly different from that in permanently blind participants. In contrast, their gait parameters did not differ significantly from those of sighted controls. The present findings highlight both the need for visual calibration of proprioceptive and vestibular systems and the crossmodal adaptability of locomotor functions.

## Introduction

To maintain postural control during upright standing and locomotion, the brain integrates signals from the motor system and sensory signals of the visual, proprioceptive, vestibular, and auditory systems (Balasubramaniam & Wing, 2002; Cullen, 2012; Forbes, Chen, & Blouin, 2018; Peterka, 2018). When input of one sensory system is missing or impaired, postural stability is decreased and gait patterns are changed (Diener, Dichgans, Guschlbauer, & Mau, 1984; Dieterich & Brandt, 2008; Henry & Baudry, 2019; Stroh, Rösler, & Röder, 2021).

The role of sight in postural control has mainly been studied in permanently blind and visually impaired individuals. Larger postural sway during upright standing (Giagazoglou et al., 2009; Rogge et al., 2021; Schmid, Nardone, De Nunzio, Schmid, & Schieppati, 2007) and altered gait patterns with slower gait velocity, shorter stride length, limited ankle plantar flexion, and prolonged duration of stance (Hallemans, Ortibus, Meire, & Aerts, 2010; Nakamura, 1997) have been reported in blind and visually impaired participants compared with normally sighted controls. Although some results have suggested that blind individuals make more extensive use of somatosensory and vestibular cues to maintain posture (Nakata & Yabe, 2001; Schieppati, Schmid, & Sozzi, 2014; Schwesig et al., 2011), they typically perform worse in static and dynamic balance tasks than normally sighted participants tested with eyes open (Campayo-Piernas, Caballero, Barbado, & Reina, 2017; Ozdemir, Pourmoghaddam, & Paloski, 2013; Ray, Horvat, Croce, Mason, & Wolf, 2008; Rogge et al., 2019; Schmid et al., 2007).

When blind individuals were compared with blindfolded normally sighted participants, no group differences or worse performance of blind participants in balance and gait performance were observed (Ozdemir et al., 2013; Rogge et al., 2019; Rogge et al., 2021; Schmid et al., 2007). These results suggest that blind individuals do not considerably compensate for the absence of vision during postural control. This is partially in contrast to the extant literature on crossmodal compensation after visual deprivation; that is, enhanced processing within the intact sensory systems. For example, congenitally blind individuals have been reported to perform better than normally sighted individuals in auditory and tactile tasks such as sound frequency and temporal discrimination, sound localization, speech perception, auditory motion perception, language comprehension, tactile discrimination, and short- and long-term memory (reviewed in Bedny, 2017; Renier, De Volder, & Rauschecker, 2014; Röder & Kekunnaya, 2022). To cope with everyday life situations, blind individuals rely more on the auditory and tactile modality than sighted individuals. Therefore, enhanced use-dependent plasticity has been discussed as a mechanism underlying crossmodal compensation after sensory deprivation (Pavani & Röder, 2012). However, when it comes to motor functions, blind children and adults have been reported to more often adopt a sedentary lifestyle than their sighted peers (Augestad & Jiang, 2015; Houwen, Hartman, & Visscher, 2008; Longmuir & Bar-Or, 2000; Müürsepp, Arjokesse, Ereline, Pääsuke, & Gapeyeva, 2018; Rogge et al., 2021). Thus, they may lack extensive motor experience to fully acquire postural control and balance abilities in the absence of vision (Schmid et al., 2007).

In typically developing children, visual and motor development are tightly linked during the first year of life. On the one hand, visual stimuli elicit exploration behavior in infants, such as reaching in 4-month-olds (von Hofsten, 1991) and locomotion when infants begin to crawl and walk (Adolph & Robinson, 2015). On the other hand, mastering motor developmental milestones enriches the infants’ visual input, from lifting the head and chest during the first months of life up to upright standing and walking at the end of the first year (Adolph & Robinson, 2015). Vision has been discussed as being important for spatial calibration of the vestibular and the proprioceptive system during early development (Prechtl, Cioni, Einspieler, Bos, & Ferrari, 2001; Röder, Kusmierek, Spence, & Schicke, 2007). As a result, poor postural control is expected if visual input is lacking during the first months of life. Results in congenitally blind individuals have indicated delays in motor development compared with typically developing children, which support this assumption (Cuturi, Aggius-Vella, Campus, Parmiggiani, & Gori, 2016; Prechtl et al., 2001; Tröster & Brambring, 1993). Nevertheless, blind children do reach major motor developmental milestones such as sitting, crawling, standing up, and walking, and they learn complex motor skills needed in daily physical activities (Nakata & Yabe, 2001).

Whether or not visual input during sensitive periods of motor–visual development is essential for later postural control has been addressed in a few studies comparing performance of congenitally blind and late blind participants in balance and gait tasks, respectively. These studies did not report any significant differences in postural stability or gait between congenitally blind and late blind participants (da Silva et al., 2018; Rogge et al., 2019; Schmid et al., 2007). Furthermore, performance of congenitally and late blind participants did not differ from sighted participants tested with eyes closed but was impaired when compared with sighted participants tested with eyes open. These findings suggest that the absence of vision during task execution is a major factor explaining variance in how participants perform. As sample sizes were small and participant samples were rather heterogeneous in these studies, the strong impact of acute vision might have obscured more subtle effects of early visual experience on postural control. Thus, attempts to disentangle the impact of visual input from the effects of visual experience in early versus late phases of visuomotor development are necessary for a better understanding of reasons why compensation in postural control is limited in blind humans. The study of congenitally cataract-reversal participants who had experienced a transient phase of visual deprivation early in life and regained sight after cataract-removal surgery offers a unique opportunity to separate the effects of early visual experience from the effects of visual input during the assessment of visuomotor functions. Moreover, comparing balance and gait between congenitally cataract-reversal individuals and permanently congenitally blind individuals allows assessing whether some recovery (that is, the use of vision) is possible in balance tasks and for gait control. Finally, comparing individuals with reversed congenital and later emerging cataracts (developmental cataract-reversal individual) provides hints about possible sensitive periods in these functional domains.

The aim of the present study was to investigate the role of visual cues and early visual experience in postural control (balance) and locomotion (gait). To this end, we assessed balance and gait in congenitally cataract-reversal (CC) individuals, developmental cataract-reversal (DC) individuals, permanently congenitally blind (CB) individuals, permanently late blind (LB) individuals, and normally sighted controls (SCs). If early developmental visual experience is important for the calibration of the vestibular and the proprioceptive systems, we expected that both SCs and DC individuals would outperform CC individuals. Moreover, LB individuals were expected to show better balance and gait performance than CB individuals. If visual input during the task is predominantly defining balance and gait, we would predict CC and DC individuals who still suffer from visual impairments to perform worse than SC individuals but better than CB and LB individuals.

Comparing CC participants with CB participants and DC participants with LB participants allowed us to estimate the extent of potential recovery of postural control after an early versus a later phase of visual deprivation. In addition, we assessed whether permanently blind participants compensate for the lack of vision. If so, we would expect them to outperform the SC group when tested with eyes closed.

## Materials and methods

### Participants

Fifty-nine individuals took part in the present study. All participants were tested at the LV Prasad Eye Institute, Hyderabad, India (LVPEI). Cataract-reversal participants were recruited at the LVPEI by ophthalmologists and optometrists. Permanently blind participants were recruited from the Institute for Vision Rehabilitation at the LVPEI. Normally sighted participants were recruited from the local community by word of mouth in the surrounding area of the eye care institute. Participants’ characteristics are summarized in Table 1.

**Table 1.** Participants’ characteristics. *Notes.* ^1^The significant main effect of age was due to LB individuals being on average older than all other groups. ^2^Higher values indicate a higher degree of visual loss. ^3^Data are missing for two participants.

	Congenital cataract	Developmental cataract	Congenitally blind	Late blind	Sighted controls	*p*
*n*	11	11	12	12	13	—
Female/male, *n*	2/9	3/8	1/11	2/10	2/11	—
Age (y), mean ± *SD*	19.55 ± 11.82	15.09 ± 4.68	20.08 ± 4.70	28.17 ± 7.00	17.77 ± 10.30	0.005^1^
Visual acuity (logMAR),^2^ mean ± *SD*	0.89 ± 0.49	0.13 ± 0.29	—	—	—	<0.001
Age at surgery (y), mean ± *SD*	6.70 ± 6.75	8.55 ± 4.13	—	—	—	0.450
Duration of blindness (y), mean ± *SD*	—	—	20.08 ± 4.70	8.63 ± 7.80	—	<0.001
Reported age at onset of visual complaints (y), mean ± *SD*	—	8.36 ± 3.41	—	12.95 ± 6.86^3^	—	0.078

The group of individuals with a history of dense bilateral congenital cataracts (CC) consisted of 11 participants (two females; mean age ± *SD* at testing, 19.55 ± 11.82 years; range, 7–43). Their cataracts were surgically removed between the ages of 5 month and 21 years, and on average 13 years (*SD* = 9.03; range, 1–30) had elapsed before balance and gait were assessed. The diagnosis of a history of congenital cataract was made by ophthalmologists and optometrists based on the presence of one or more of the following symptoms as documented in the medical records: (a) lack of pattern vision after birth as reported by the participant or a guardian; (b) medical records confirming a lack of fundus visibility prior to surgery due to opacified lenses; (c) documentation of the presence of nystagmus prior to surgery and persistent nystagmus after surgical removal of the opaque lenses; (d) presence of strabismus; or (e) positive family history of congenital cataracts. Visual acuity in CC individuals was measured with the Freiburg Visual Acuity Test (FrACT) (Bach, 1996) using Landolt-C stimuli at the day of balance testing (*n* = 3) or the closest clinical visit (*n* = 8). The average time between visual acuity testing and assessments of postural control was 23.5 days. CC individuals had a mean logMAR visual acuity of 0.89 (*SD* = 0.49; range 0.03–1.76). Positive logMAR values indicate the degree of vision loss.

In addition, 11 individuals with a history of developmental cataracts (DC) were tested (three females; mean age 15.09 ± 4.68 years; range 9–24). Participants of the DC group had pattern vision after birth and reported the onset of visual complaints after the age of 2 years (age range for onset of visual complaints 2–12 years). Developmental cataracts were surgically removed between the ages of 2 and 17 years, and on average 6.55 years (*SD* = 5.61; range 1–22) had elapsed before balance and gait were assessed. Clinical diagnosis of developmental cataracts was confirmed by the reported age of onset of visual complaints being later than 1 year of age, lack of nystagmus, and the absence of a positive family history of congenital cataracts. Visual acuity was measured at the day of testing (*n* = 5) or during the closest clinical visit (*n* = 6). The average time between visual acuity testing and assessments of postural control was 7.7 days. DC individuals had a mean logMAR visual acuity of 0.13 (*SD* = 0.29; range −0.23 to 0.63).

Moreover, two groups of permanently blind individuals were assessed. Blindness was due to peripheral reasons in all cases. Twelve participants were born blind (CB; one female; mean age 20.08 ± 4.70 years; range 12–27). Twelve participants experienced vision in early life and permanently lost vision after the age of 3 years (LB; two females; mean age 28.17 ± 7.00 years; range 20–41; mean age at onset of blindness 19.54 years; age range for onset of blindness 3–37 years).

Sight-recovery individuals and permanently blind individuals were compared with a group of 13 sighted controls (SCs) who reported typical visual development and who had normal or corrected-to-normal vision at the time of assessment. SC participants were recruited to best match the CC group in terms of age and gender distribution (two females; mean age 17.77 ± 10.30 years; range 8–40).

None of the participants reported a history of neurological disorders, impairments in other sensory systems or acute orthopedic problems. Written informed consent was obtained from all participants or, for minors, from a legal guardian. CC and LB participants, as well as participants who were not proficient in English, were verbally informed of the details of the study protocol, privacy protection, and their rights to discontinue assessments and withdraw consent at any time in a language they could fully understand. Adults received monetary compensation for the time spent in the study and for travel expenses associated with the study. In the case of minors, the compensation was given to the parents or legal guardians for lost wages; minor participants received a small present. Both the Institutional Ethics Review Board of LV Prasad Eye Institute (Hyderabad, India) and the local ethics board of the Faculty of Psychology and Human Movement at the University of Hamburg (Hamburg, Germany) approved the present study. The study adhered to the tenets of the Declaration of Helsinki (World Medical Association, 2013).

### Assessments

The assessments of upright standing balance and gait control followed the protocol as described in Rogge et al. (2021).

#### Single-leg stance time

Balance performance was assessed with barefoot single-leg stances with eyes open and eyes closed. The underground was either a firm surface (hard ground) or a 10-cm flat cushion of medium density foam (soft ground; Airex Balance Pad; Gaugler & Lutz, Aalen, Germany). Participants were asked to place their hands on their hips and to lift their dominant foot with their head straight-ahead. To determine the dominant foot, participants were asked to imagine kicking a ball and indicate which foot they would use to do so.

Each trial had a maximum length of 60 seconds, followed by some resting time. A trial ended when a participant touched the floor with the lifted foot, rotated or moved the foot of the standing leg to maintain balance, removed hands away from the hips, or grabbed nearby objects. Twelve trials were run for each CC, DC, and SC participant, three for each of the four conditions (hard ground/eyes open, soft ground/eyes open, hard ground/eyes closed, and soft ground/eyes closed). Trials with eyes open were run first, followed by trials with eyes closed. Participants were blindfolded during the eyes-closed conditions with an eye mask. The order of hard and soft ground was counterbalanced across participants. CB and LB groups performed a total of six trials, three for each condition (hard and soft ground). Balance data were missing for one LB participant. The mean time (in seconds) per condition that the participant remained in the correct position was used as the dependent variable.

#### Gait parameters

Gait parameters were captured with a wireless motion tracker (MTw sensors, sampling rate, 100 Hz; Xsens Technologies, Enschede, The Netherlands) attached to the left foot of participants. During testing, participants walked up and down a hallway of approximately 25 meters for 5 minutes at their preferred walking speed. CC, DC, and SC individuals performed the task twice, once with eyes open and once blindfolded.

During the blindfolded condition and for CB and LB participants, two experimenters walked next to the participants and asked them to turn around at the end of the hallway. Whenever participants lost their path, they were guided back toward the correct path. One DC participant was not able to perform the gait task with eyes closed and was, therefore, not included in any analysis of gait parameters. Data for the first and the last 25-m bouts and for the first and the last 2.5 meters of each 25-meter bout were excluded. Furthermore, the kinematic time series were visually checked. Areas with non-stationary data (e.g., when a participant stopped and was guided back on the correct way) were excluded from data analyses. The parameters of stride length, stride time, gait speed, and minimum foot clearance, as well as the variability of each parameter (intra-individual standard deviation), were determined. The reliability of the system (inertial sensors and algorithms) has been verified (Hamacher, Hamacher, Taylor, Singh, & Schega, 2014). For estimating gait variability measures, previous work has recommended the inclusion of at least 50 strides (Konig, Singh, von Beckerath, Janke, & Taylor, 2014). Based on this criterion, we excluded the gait data of four participants (two CB and twoLB).

Furthermore, the largest divergence exponent (LDE) as a measure of local dynamic gait stability was calculated based on three-dimensional angular velocity data of the foot (Hamacher, Hamacher, Singh, Taylor, & Schega, 2015). To estimate gait stability (LDE), the same number of strides for all participants had to be included in the analysis, with a higher number of strides (up to 150 strides) leading to more precise estimates (Bruijn, van Dieen, Meijer, & Beek, 2009). Because the number of strides varied among participants, there was a trade-off between measurement precision and the number of participants included. The number of strides per participants ranged from 61 to 238 strides (mean number of strides 138 ± 34). Four participants (5% of the sample: one CC, two CB, and one LB) had much lower stride counts compared with the remaining sample and were therefore additionally excluded from the LDE analysis to improve measurement precision. For the included participants, the middle 89 strides per condition were analyzed. To compute LDE, we time-normalized the three-dimensional angular velocity data of 89 strides (minimum across participants and conditions) to 8900 samples. Using the delayed embedding approach, we chose the time delay (τ = 12, mean across participants and conditions) and the embedded dimension (dE = 15, minimum across participants and conditions) based on the first minimal mutual information (Fraser & Swinney 1986) and the false nearest neighbor analysis (Kennel, Brown, & Abarbanel, 1992), respectively. Based on the resulting state–space, the short-term LDE was calculated using the algorithm of Rosenstein, Collins, and de Luca (1993) with a time range of 50 samples (0.5 strides on average). Higher LDE values were interpreted as lower local dynamic gait stability and vice versa. Gait variability parameters and LDE have been shown to depict reasonable construct and convergent validity to assess gait stability (e.g., as a measure of the probability of falling) (Bruijn, Meijer, Beek, & van Dieën, 2013).

### Data analysis

Data were analyzed in R 4.1.2 (R Core Team, 2021) using the R packages plyr, dplyr, tidyr, tidyverse, ggplot2, sjstats, afex, emmeans, and pwr (R Foundation for Statistical Computing, Vienna, Austria). Balance performance was compared between sight-recovery participants and sighted controls by means of a mixed-design three-way analysis of variance (ANOVA) with the between-subject factor group (CC, DC, SC), the within-subject factors vision (eyes open, eyes closed) and condition (hard ground, soft ground), and the dependent variable seconds in the correct position. Significant three-way interactions were followed by two-way ANOVAs with the between-subject factor group (CC, DC, SC) and the within-subject factor condition (hard ground, soft ground), separately for the eyes-open and eyes-closed conditions. For gait parameters, the mixed-design two-way ANOVA had the between-subject factor group (CC, DC, SC) and the within-subject factor vision (eyes open, eyes closed). Separate analyses were run for the dependent variables stride length, stride length variability, stride time, stride time variability, gait speed, gait speed variability, minimum toe clearance, minimum toe clearance variability, and local dynamic gait stability (LDE).

Furthermore, balance performance in the eyes-closed condition was compared among participant groups by means of a mixed-design, two-way ANOVA with the between-subject factor group (CC, DC, CB, LB, SC) and the within-subject factor condition (hard ground, soft ground). For gait parameters, a one-way ANOVA was run with the between-subject factor group (CC, DC, CB, LB, SC). Statistical comparisons utilized Type III sums of squares. Post hoc comparisons between groups used estimated marginal means with Tukey's correction for multiple comparisons.

The normality assumption for the ANOVA models was tested by visually inspecting the residuals using *Q*-*Q* plots. We consider the detectable violations as negligible given that the ANOVA is known to be robust against violations of the normal distribution assumption (e.g., Harwell, Rubinstein, Hayes, & Olds, 1992; Schmider, Ziegler, Danay, Beyer, & Bühner, 2010). In addition, we ran non-parametric tests to show the robustness of the present results. The non-parametric Kruskal–Wallis-test was employed for the one-way ANOVA and parametric bootstrapping for mixed-effects ANOVAs. The additional analyses confirmed the pattern of results (see Supplementary Materials). Pearson correlation coefficients were calculated to assess associations between visual acuity and balance and gait parameters, respectively, in sight-recovery participants. Effects at *p* < 0.05 were considered significant.

## Results

### Balance performance: Single-leg stance

#### Sight-recovery participants and normally sighted controls, eyes open and eyes closed

**Figure 1.**
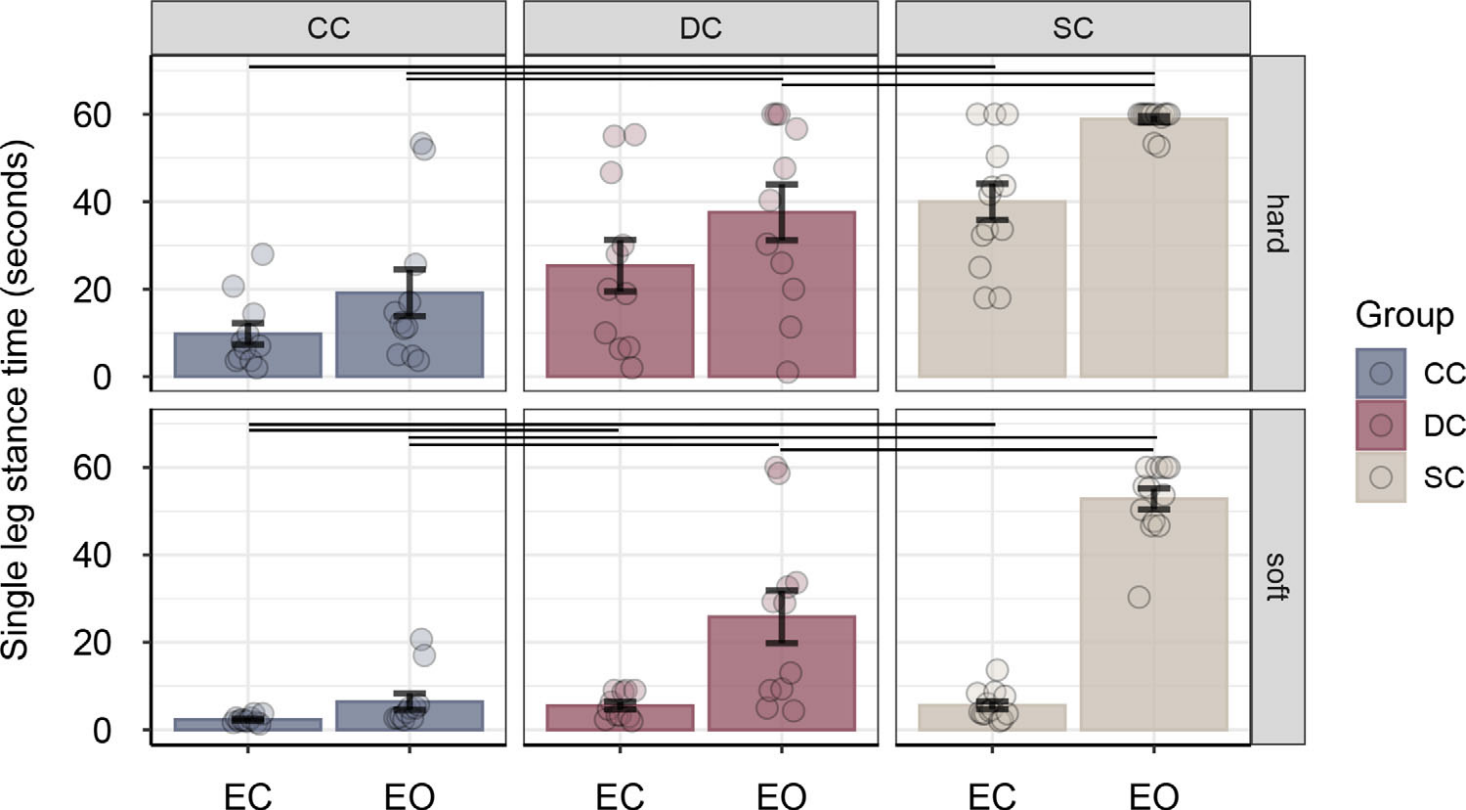
Mean single-leg stance time (in seconds) for sight-recovery participants (CC and DC) and normally sighted controls (SCs), separately for the eyes-closed (EC) condition versus the eyes-open (EO) condition and for hard versus soft ground. Error bars show standard errors of the mean. Dots represent data for individual participants. Horizontal lines indicate significant pairwise group differences (*p* < 0.05, post hoc test).

Overall, balance performance was better in the eyes-open condition compared with the eyes-closed condition, main effect of vision *F*(1, 32) = 100.81, *p* < 0.001, η^2^*_G_* = 0.373, and better on hard ground compared with soft ground, main effect of condition *F*(1, 32) = 71.646, *p* < 0.001, η^2^*_G_* = 0.288. Comparing balance performance between the sight-recovery groups and normally sighted controls revealed that the time participants were able to stand on one leg was shortest for CC participants and longest for SC participants, with the performance of the DC group falling in between, main effect of group *F*(2, 32) = 31.790, *p* < 0.001, η^2^*_G_* = 0.512 (Figure 1). The three-way interaction of group, vision, and condition was significant, indicating that differences between groups depended on visual input and testing condition, *F*(2, 32) = 13.348, *p* < 0.001, η^2^*_G_* = 0.078. To further explore the three-way interaction, separate two-way ANOVAs with the within-subject factor condition and the between-subject factor group were calculated for the eyes-closed and eyes-open conditions, respectively. When tested with eyes open, all groups performed better on hard ground compared with soft ground, main effect of condition, *F*(1, 32) = 20.17, *p* < 0.001, η^2^*_G_* = 0.122, with no significant condition × group interaction, *F*(2, 32) = 0.882, *p* = 0.424, η^2^*_G_* = 0.012. The main effect of group was significant, *F*(2, 32) = 35.312, *p* < 0.001, η^2^*_G_* = 0.632. Post hoc contrasts revealed that CC and DC participants performed worse than SC participants, on both hard ground and soft ground, all |*q_s_*(32)| > 3.34, all *p* < 0.006, all |*d*| > 1.35. Moreover, single-leg stance times were significantly shorter for CC participants compared with DC participants on both hard and soft ground, all |*q_s_*(32)| > 2.78, *p* < 0.024, all |*d*| > 0.94. When the participants were tested with eyes closed, the main effect of group, *F*(2, 32) = 11.88, *p* < 0.001, η^2^*_G_* = 0.312; the main effect of condition, *F*(1, 32) = 81.85, *p* = 0.001, η^2^*_G_* = 0.500; and the interaction between group and condition reached significance, *F*(2, 32) = 12.126, *p* < 0.001, η^2^*_G_* = 0.228. Post hoc contrasts revealed that CC participants performed worse than SC participants on both hard and soft ground, all |*q_s_*(32)| > 3.04, all *p* < 0.013, all |*d*| > 1.43]; whereas, the lower performance of CC participants compared with DC participants reached significance on soft ground only, |*q_s_*(32)| = 2.63, *p* = 0.020, |*d*| = 1.48.

**Figure 2.**
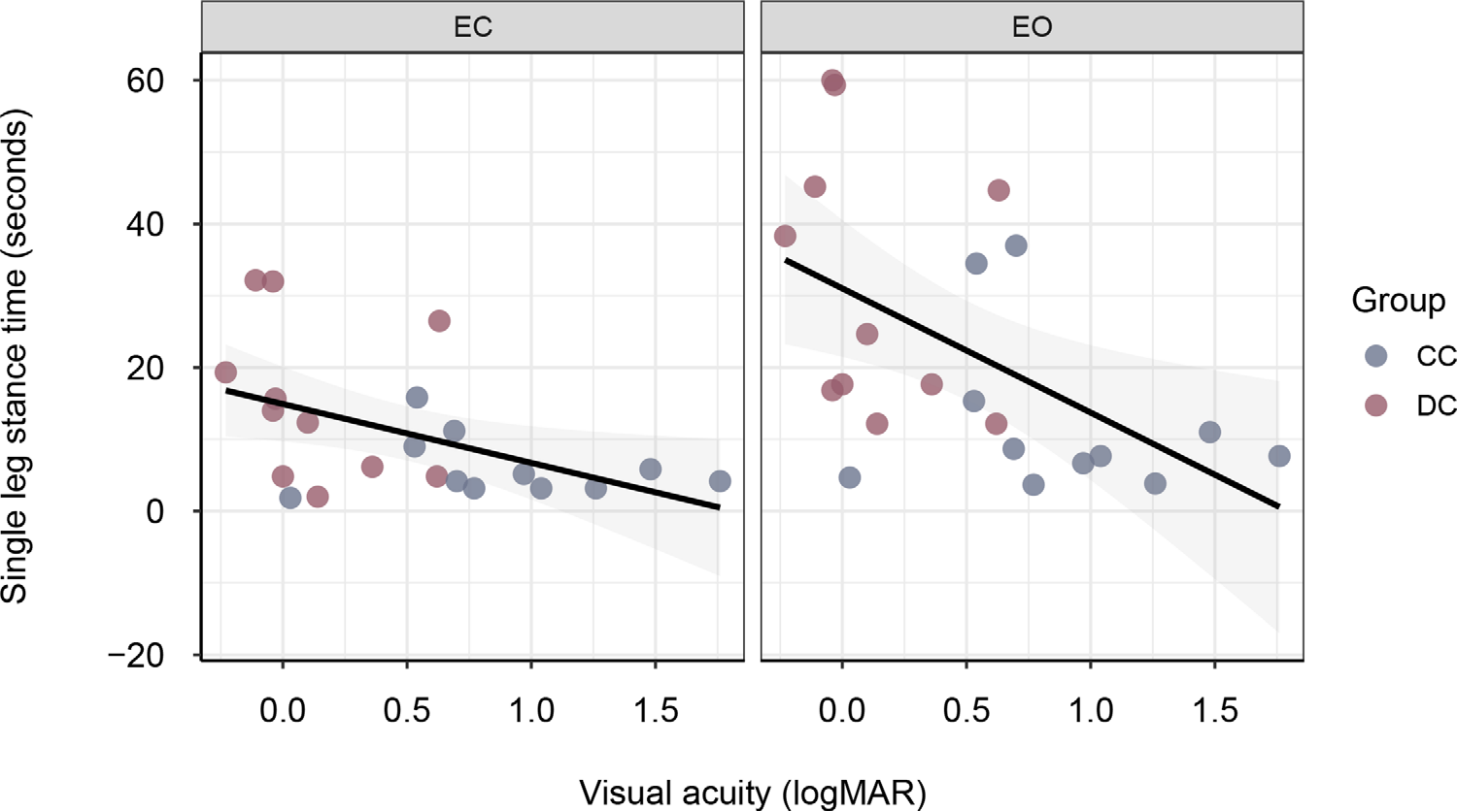
Association between visual acuity (in logMAR) and single-leg stance time (in seconds) in sight-recovery participants, collapsed across hard and soft ground. Positive logMAR values indicate the degree of vision loss; higher values denote lower visual acuity.

Balance performance in sight-recovery participants was associated with visual acuity; that is, the higher participants’ visual acuity, the longer were their single-leg stance times. This was found both for the eyes-open condition, *r*(20) = –0.53, *p* = 0.011, and eyes-closed condition, *r*(20) = –0.48, *p* = 0.024 (Figure 2).

#### All participant groups, eyes closed

**Figure 3.**
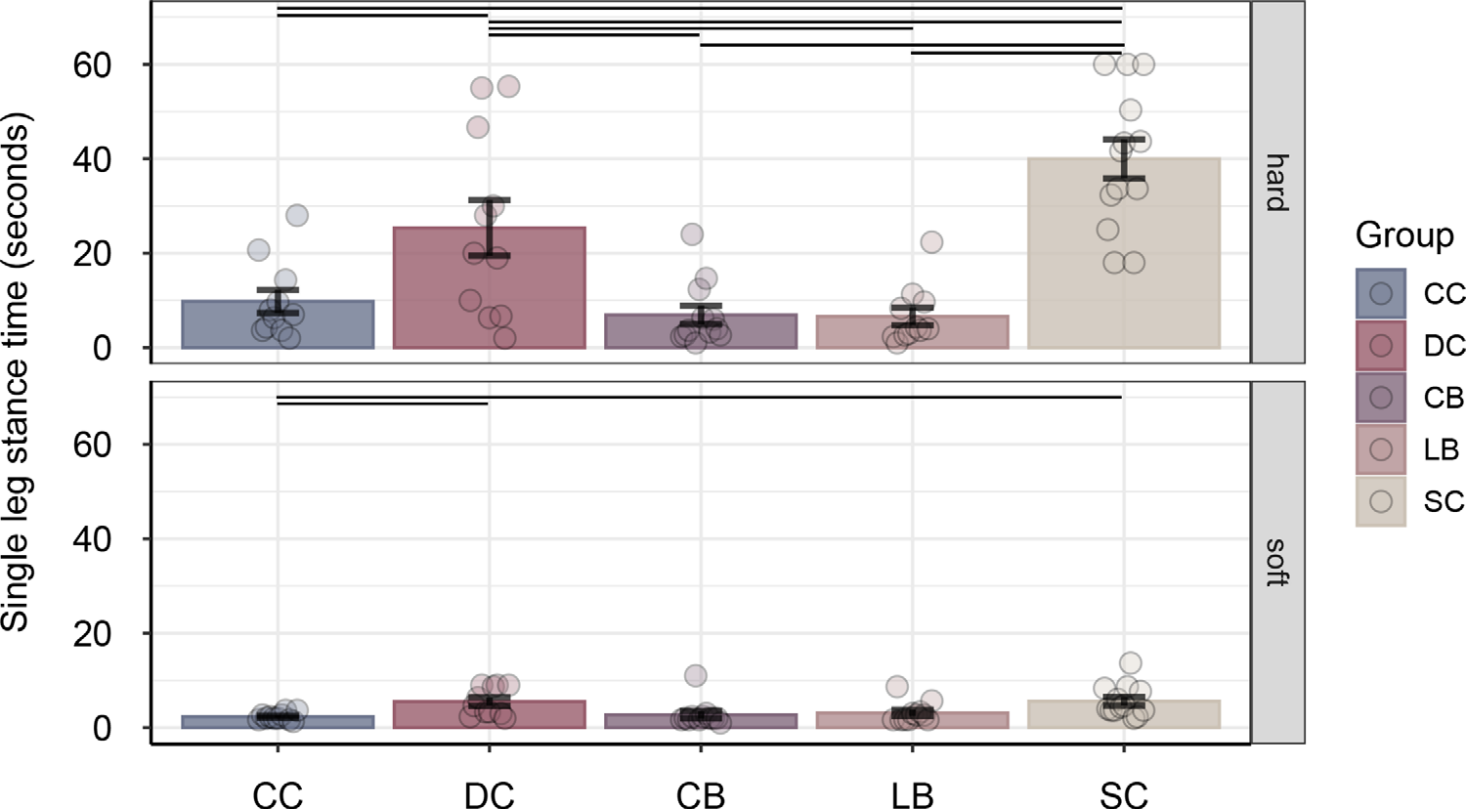
Mean single-leg stance time (in seconds) with eyes closed, separately for congenitally cataract-reversal (CC) participants, developmental cataract-reversal (DC) participants, permanently congenitally blind (CB) participants, permanently late blind (LB) participants, and normally sighted controls (SCs) and for hard versus soft ground. Error bars show standard errors of the mean. Dots represent data for individual participants. Horizontal lines indicate significant pairwise group differences (*p* < 0.05, post hoc test).

The single-leg stance task was easier on hard ground compared with soft ground, main effect condition *F*(1, 53) = 86.85, *p* < 0.001, η^2^*_G_* = 0.399. Performance differences between groups were more pronounced on hard ground than on soft ground, group × condition *F*(4, 53) = 16.83, *p* < 0.001, η^2^*_G_* = 0.339 (Figure 3). The time participants were able to stand on one leg on hard ground was significantly lower for CC, CB, and LB individuals compared with DC and SC individuals, all |*q_s_*(53)| > 2.967, all *p* < 0.035, all |*d*| > 1.04. Moreover, the SC group showed better performance than the DC group, |*q_s_*(53)| = 2.90, *p* = 0.042, |*d*| = 0.83. There were no significant differences among the CC, CB, and LB groups, all |*q_s_*(53)| < 0.61, all *p* > 0.973, all |*d*| < 0.45. On soft ground, CC participants performed worse compared with DC and SC participants, all |*q_s_*(53)| > 2.93, all *p* < 0.039, all |*d*| > 1.43. Moreover, CB participants tended to show reduced performance compared with DC individuals, |*q_s_*(53)| = 2.63, *p* = 0.080, |*d*| = 1.00, and with SC individuals, |*q_s_*(53)| = 2.81, *p* = 0.051, |*d|* = 0.97.

### Gait parameters

#### Sight-recovery participants and normally sighted controls, eyes open and eyes closed

**Figure 4.**
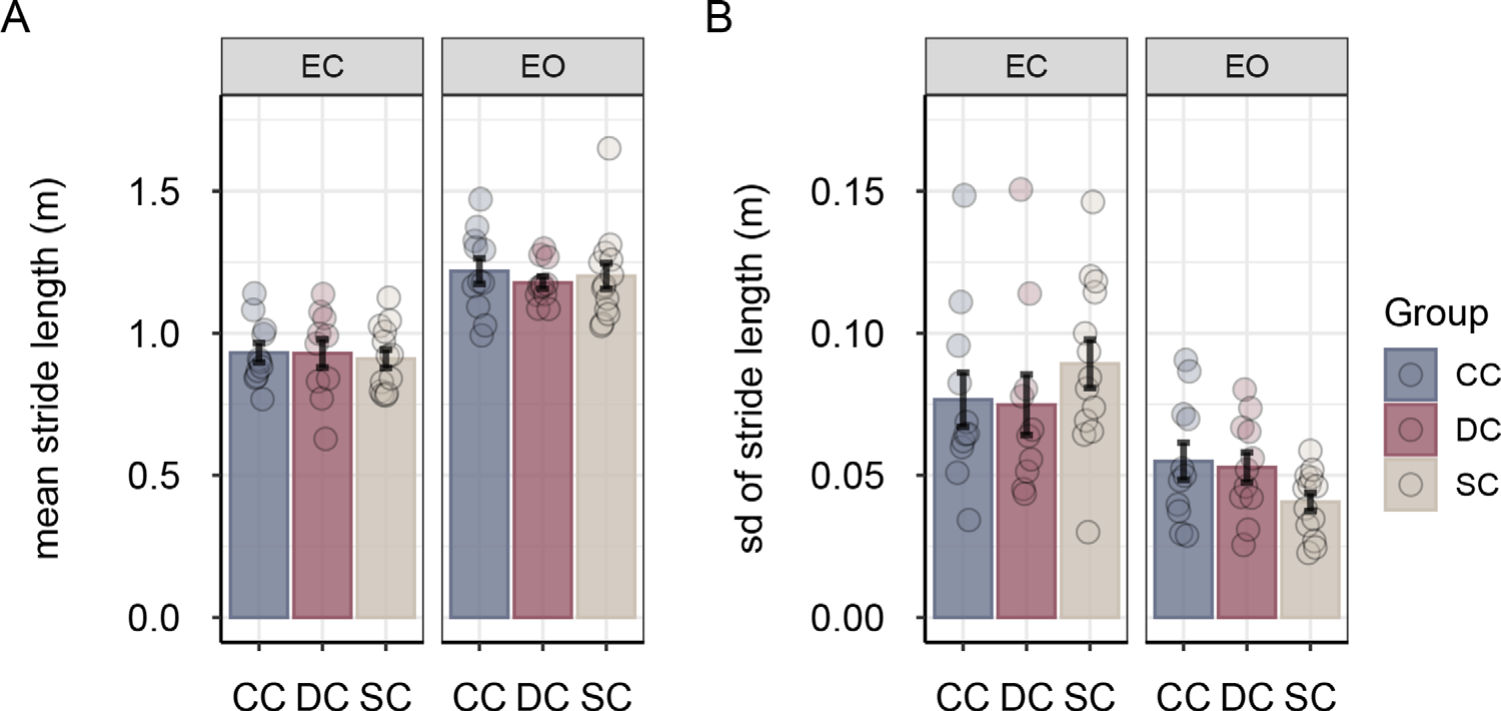
Mean stride length (**A**) and mean variability of stride length (**B**) for sight-recovery participants (CC and DC) and normally sighted controls (SCs), separately for the eyes-closed and eyes-open conditions. Error bars show standard errors of the mean. Dots represent data for individual participants.

**Figure 5.**
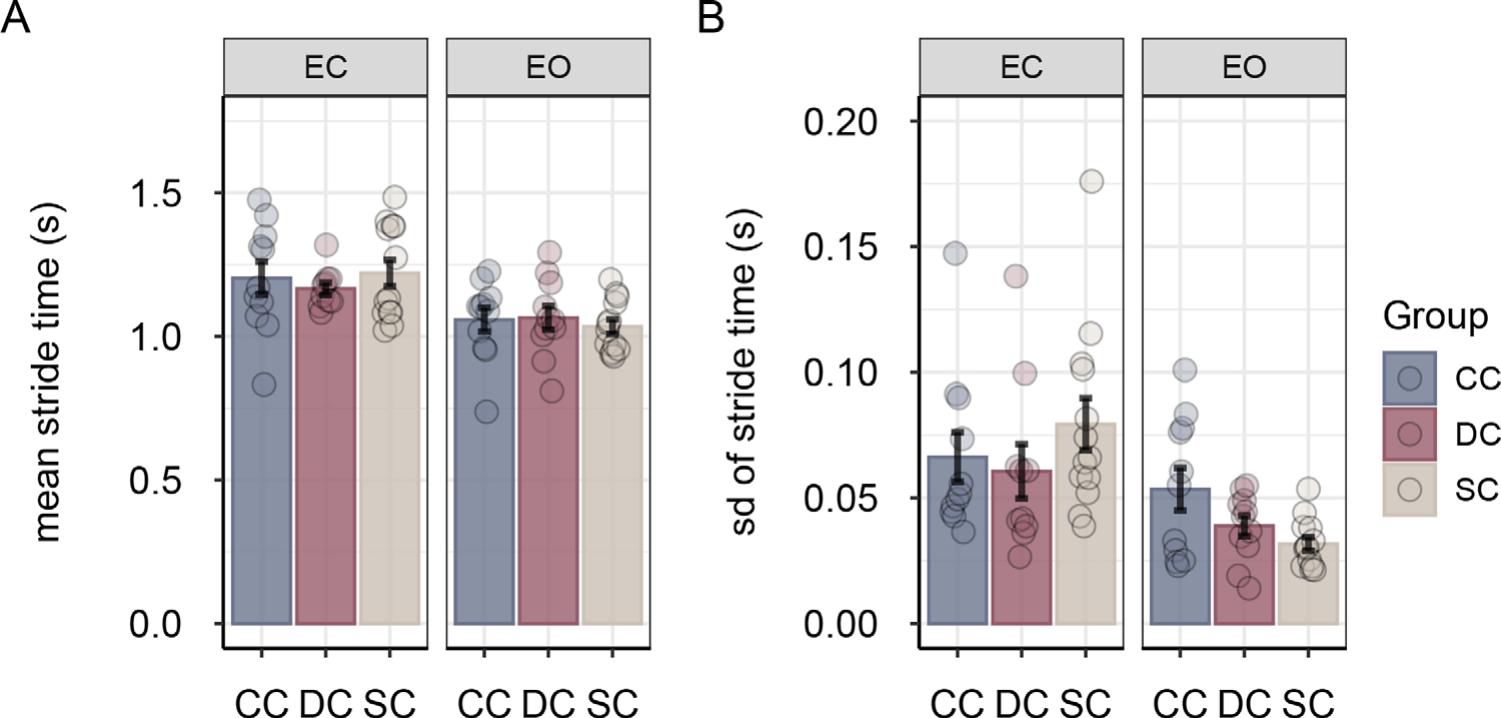
Mean stride time (**A**) and mean variability of stride time (**B**) for sight-recovery participants (CC and DC) and normally sighted controls (SCs), separately for the eyes-closed and eyes-open conditions. Error bars show standard errors of the mean. Dots represent data for individual participants.

Participants made longer strides and stride length was less variable in the eyes-open condition compared with the eyes-closed condition, main effect of vision *F*(1, 31) = 118.89, *p* < 0.001, η^2^*_G_* = 0.531 for stride length; *F*(1, 31) = 24.68, *p* < 0.001, η^2^*_G_* = 0.296 for variability of stride length (Figure 4). Moreover, stride time was significantly shorter and less variable in the eyes-open condition than in the eyes-closed condition, main effect of vision *F*(1, 31) = 52.92, *p* < 0.001, η^2^*_G_* = 0.222 for stride time; *F*(1, 31) = 14.42, *p* < 0.001, η^2^*_G_* = 0.212 for variability of stride time (Figure 5). Groups did not differ in stride length and stride time, and there were no significant group × vision interactions for any of the stride parameters (all *F* < 2.20, all *p* > 0.13, all η^2^*_G_* < 0.08).

**Figure 6.**
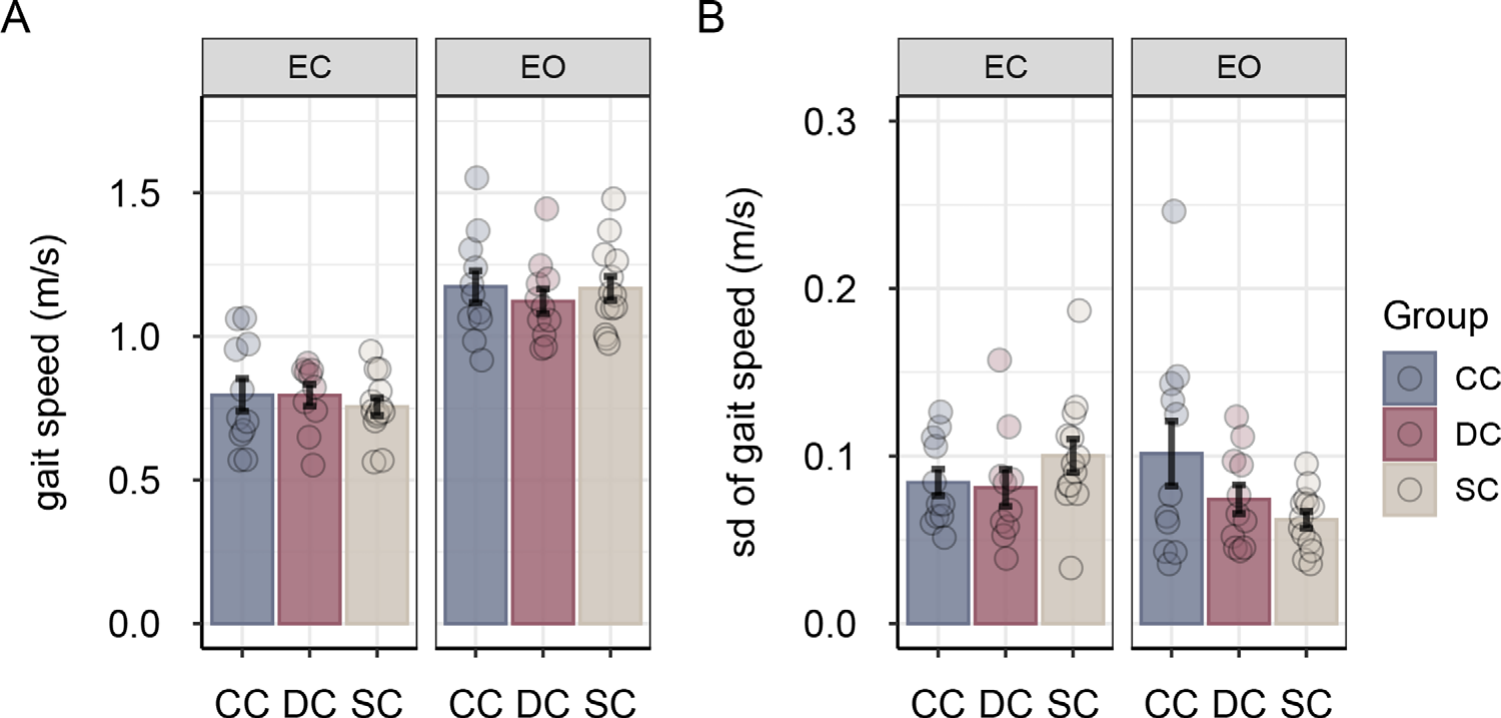
Mean gait speed (**A**) and mean variability of gait speed (**B**) for sight-recovery participants (CC and DC) and normally sighted controls (SCs), separately for the eyes-closed and eyes-open conditions. Error bars show standard errors of the mean. Dots represent data for individual participants.

Participants walked faster in the eyes-open condition compared with the eyes-closed condition, main effect of vision *F*(1, 31) = 100.99, *p* < 0.001, η^2^*_G_* = 0.615 (Figure 6A), but groups did not differ in mean gait speed (main effects of group and group × vision interactions all *F* < 0.60, all *p* > 0.50, all η^2^*_G_* < 0.02). The variability of gait speed differed between groups in the eyes-open condition but not in the eyes-closed condition, group × vision *F*(2, 31) = 4.45, *p* = 0.020, η^2^*_G_* = 0.099). Post hoc tests for the eyes-open condition revealed that CC participants tended to show more variability in gait speed compared with SC participants, *q_s_*(31) = 2.39, *p* = 0.058, *d* = 0.81 (Figure 6B)

Sight-recovery participants did not differ significantly from normally sighted controls in mean minimum toe clearance, variability of minimum toe clearance, or local dynamic gait stability (LDE) (main effects of group and group × vision interactions all *F* < 2.7, all *p* > 0.10, all η*_G_* < 0.04). Gait parameters in sight-recovery participants did not significantly correlate with visual acuity, all *r* < |0.41|, all *p* > 0.07.

#### All participant groups, eyes closed

**Figure 7.**
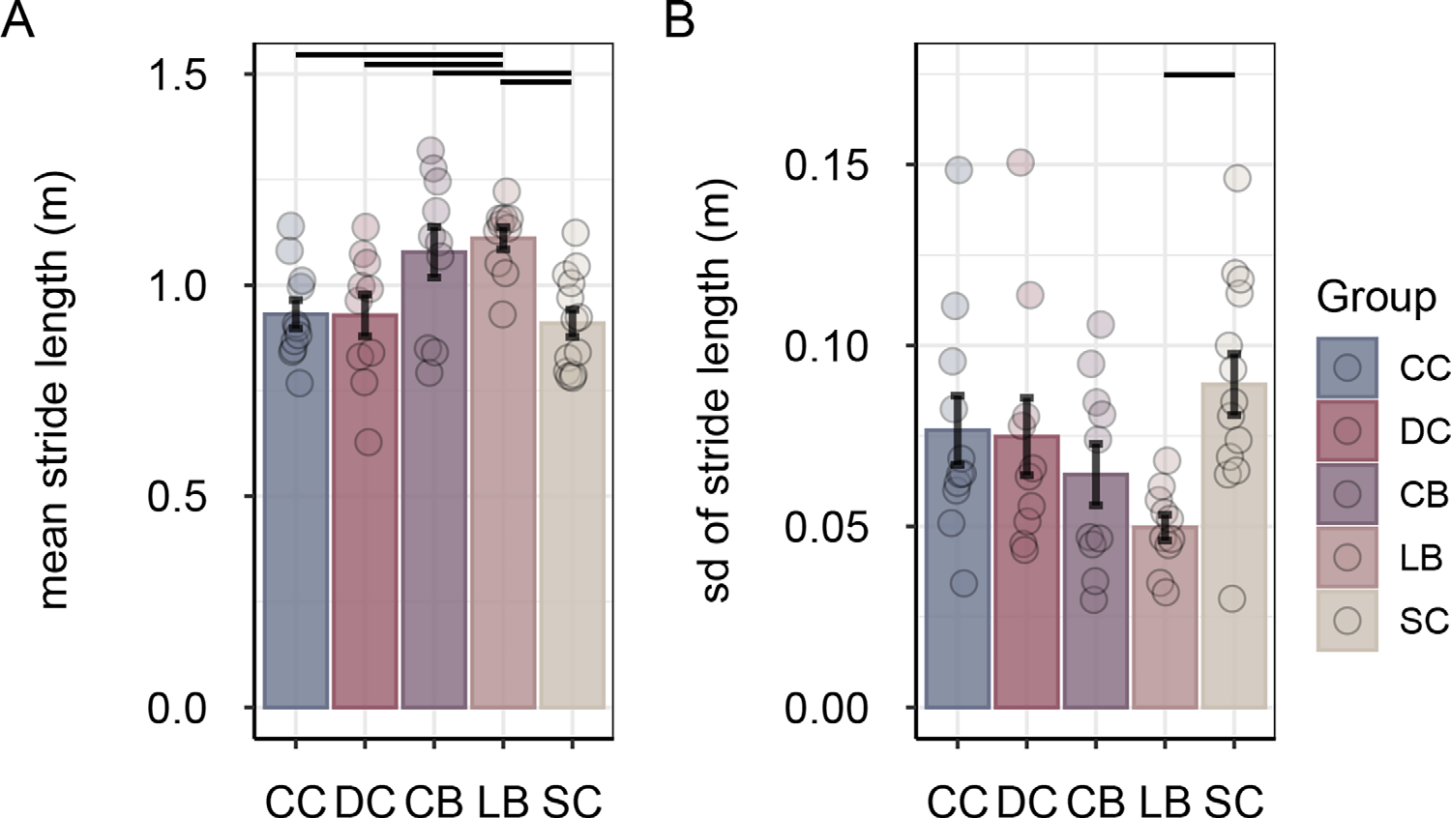
Mean stride length (**A**) and mean variability of stride length (**B**) in the eyes-closed condition, separately for congenitally cataract-reversal (CC) participants, developmental cataract-reversal (DC) participants, permanently congenitally blind (CB) participants, permanently late blind (LB) participants, and normally sighted controls (SCs). Error bars show standard errors of the mean. Dots represent data for individual participants. Horizontal lines indicate significant pairwise group differences (*p* < 0.05, post hoc test).

Permanently blind participants, both CB and LB, showed longer mean stride lengths compared with sight-recovery participants and normally sighted controls when walking with eyes closed, *F*(4, 49) = 5.22, *p* = 0.001, η^2^*_G_* = 0.299 (Figure 7A). Post hoc tests showed significant pairwise differences between the CB group compared with the SC group and between the LB group compared with the CC, DC, and SC groups, all |*q_s_*(49)| > 2.95, all *p* < 0.04, all |*d*| > 1.03. Stride length did not significantly differ between sight-recovery participants and normally sighted participants, and there was no difference between the CC and DC groups, all |*q_s_*(49)| < 0.55, all *p* > 0.90, all |*d*| < 0.19.

In addition, the variability of stride length differed among groups, *F*(4, 49) = 3.05, *p* = 0.025, η^2^*_G_* = 0.199 (Figure 7Β). LB individual showed less variability in stride length compared with SC individuals, |*q_s_*(49)| = 3.33, *p* = 0.014, *d* = 1.81. There were no significant differences among CC, DC, CB, and SC participants, all |*q_s_*(49)| < 2.18, all *p* > 0.21, all |*d*| < 0.87.

**Figure 8.**
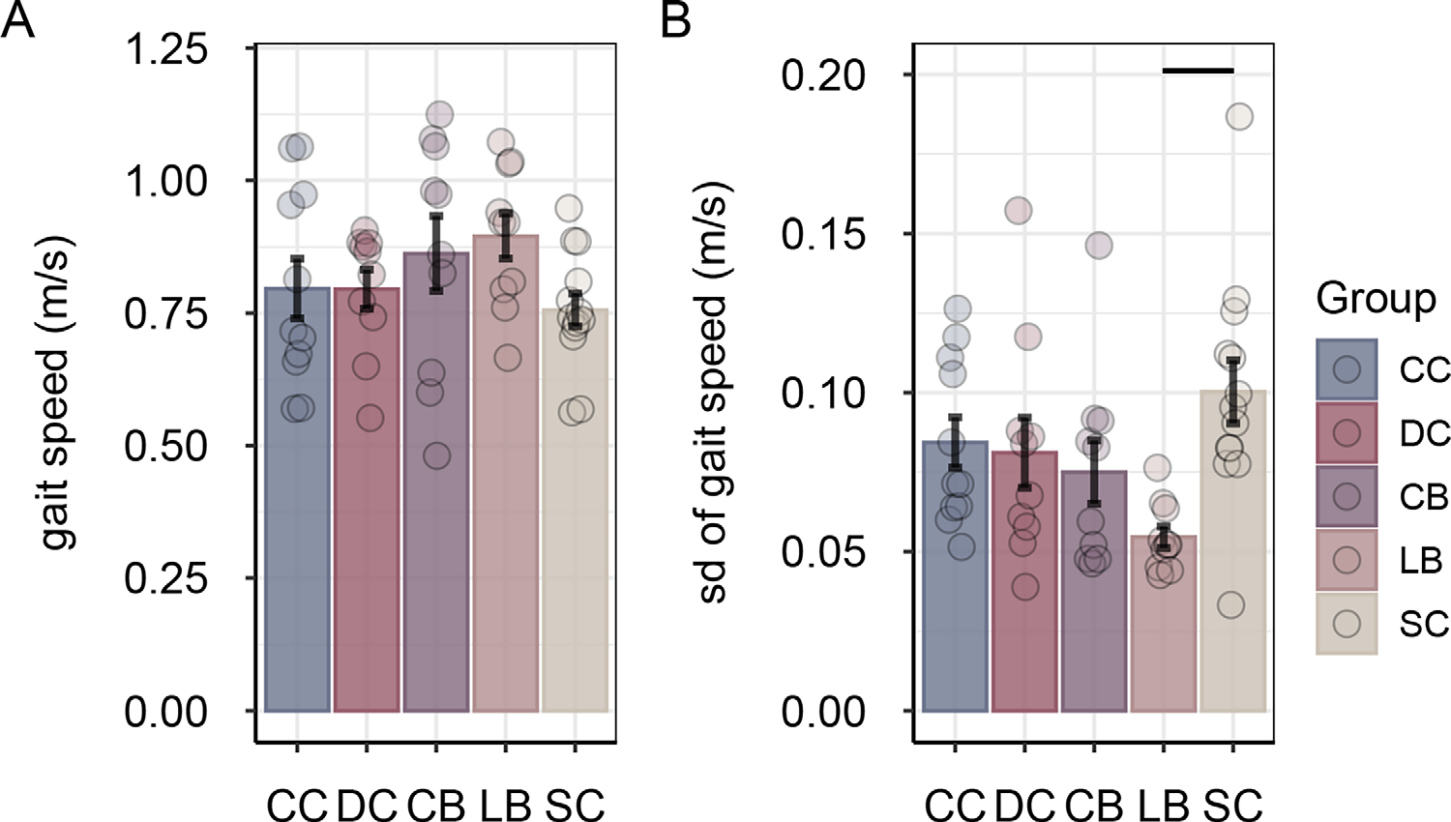
Mean gait speed (**A**) and mean variability of gait speed (**B**) in the eyes-closed condition, separately for congenitally cataract-reversal (CC) participants, developmental cataract-reversal (DC) participants, permanently congenitally blind (CB) participants, permanently late blind (LB) participants, and normally sighted controls (SCs). Error bars show standard errors of the mean. Dots represent data for individual participants. Horizontal lines indicate significant pairwise group differences (*p* < 0.05, post hoc test).

Groups did not significantly differ in gait speed, *F*(4, 49) = 1.39, *p* = 0.251, η^2^*_G_* = 0.102 (Figure 8A). LB participants showed less variability in gait speed than SC participants, main effect of group *F*(4, 49) = 3.49, *p* = 0.014, η^2^*_G_* = 0.222; post hoc contrast LB versus SC, |*q_s_*(49)| = 3.66, *p* = 0.005, |*d*| = 1.83 (Figure 8B).

Groups did not significantly differ in stride time, minimum toe clearance, or local dynamic gait stability (LDE), neither in mean values nor in the variability of these gait parameters (all *F* < 1.61, all *p* > 0.18, all η^2^*_G_* < 0.13). Descriptive statistics for all assessed gait parameters in the eyes-closed condition are reported in Table 2.

**Table 2.** Gait parameters in the eyes-closed condition for congenital cataract-reversal (CC) participants, developmental cataract-reversal (DC) participants, congenitally permanently blind (CB) participants, late permanently blind (LB) participants, and normally sighted controls (SCs). *Notes.* ^1^Data for four participants (one CC, two CB, and one LB) were not analyzed for local dynamic stability measures due to fewer than 89 strides. Lower values indicate better local dynamic gait stability.

		CC (*n* = 11)	DC (*n* = 10)	CB (*n* = 10)	LB (*n* = 10)	SC (*n* = 13)
Stride length (m)	Mean	0.93	0.93	1.08	1.11	0.91
	*SD*	0.11	0.16	0.19	0.08	0.12
Stride length variability (m)	Mean	0.08	0.07	0.06	0.05	0.09
	*SD*	0.03	0.03	0.03	0.01	0.03
Stride time (s)	Mean	1.20	1.17	1.29	1.26	1.22
	*SD*	0.19	0.07	0.19	0.16	0.17
Stride time variability (s)	Mean	0.07	0.06	0.08	0.05	0.08
	*SD*	0.03	0.03	0.05	0.02	0.04
Gait speed (m/s)	Mean	0.80	0.80	0.86	0.90	0.76
	*SD*	0.19	0.12	0.22	0.13	0.11
Gait speed variability (m/s)	Mean	0.09	0.08	0.07	0.05	0.10
	*SD*	0.03	0.03	0.03	0.01	0.04
Minimum toe clearance (mm)	Mean	12.9	12.6	12.3	12.4	13.4
	*SD*	5.6	4.9	3.7	5.3	3.1
Minimum toe clearance variability (mm)	Mean	3.7	3.5	3.4	3.0	3.6
	*SD*	1.2	1.0	0.6	0.6	0.5
Local dynamic stability (LDE)^1,2^	*n*	10	10	8	9	13
	Mean	1.19	1.16	1.31	1.27	1.19
	*SD*	0.12	0.14	0.19	0.17	0.14

## Discussion

The present study investigated the impact of visual experience on the development of postural control. In particular, we assessed whether visual deprivation during early phases of visuomotor development has long-lasting effects on postural control and locomotion. To this end, we measured balance and gait in congenitally cataract-reversal (CC) individuals, developmental cataract-reversal (DC) individuals, congenitally blind (CB) individuals, late blind (LB) individuals, and normally sighted controls (SCs). Cataract-reversal participants showed worse balance performance as indicated by shorter single-leg stance times compared with SC participants. Lower performance in this task was more pronounced in CC individuals than in DC individuals. When participants were tested with eyes closed, single-leg stance times in CC individuals did not differ from those in CB and LB individuals, and all three groups (CC, CB, and LB) performed worse than SC individuals. Moreover, balance performance correlated with visual acuity in sight-recovery individuals: The better a participant’s visual acuity, the longer they were able to stand on one leg, regardless of whether they were tested with eyes open or eyes closed. In contrast, gait parameters of sight-recovery participants did not significantly differ from SC participants. Permanently blind participants, both CB and LB participants, made longer strides compared with SC participants when the latter walked with eyes closed. Moreover, LB participants showed less variability in stride length and gait speed compared with SC participants.

### Effects of permanent and transient phases of blindness on postural control

Previous studies on the role of vision for postural control assessed permanently blind participants. Results showed larger postural sway, reduced gait speed, shorter stride length, and worse balance performance in blind participants compared with normally sighted participants tested with eyes open (Giagazoglou et al., 2009; Hallemans et al., 2010; Nakamura, 1997; Ozdemir et al., 2013; Rogge et al., 2019). When permanently blind individuals were compared with blindfolded sighted controls, some studies reported a pattern of results in blind participants similar to that in sighted participants (Ozdemir et al., 2013; Schmid et al., 2007), whereas others found worse performance of blind participants in balance performance when compared with blindfolded sighted participants (Giagazoglou et al., 2009; Rogge et al., 2021). On hard ground, we replicated the findings of lower balance performance in CB and LB individuals compared with blindfolded SC individuals, suggesting that permanently blind individuals did not compensate for the lack of vision for postural control.

In previous studies comparing permanently blind individuals to sighted individuals tested with eyes open, it was not possible to disentangle the effects of visual input during balance testing from the impact of early visual experience. In the present study, we provided evidence that individuals who regained sight by congenital or developmental cataract-removal surgery displayed higher balance scores with eyes open than with eyes closed, indicating a use of vision and thus some recovery. This is in line with recent data by Senna, Piller, Ben-Zion, and Ernst (2022) who assessed the ability of CC individuals to calibrate hand pointing to prism manipulated visual input. Senna et al. (2022) found that sensorimotor recalibration in CC individuals was impaired right after surgery but improved gradually the longer sight had been restored, suggesting that cataract-reversal individuals gained from visual input for sensorimotor recalibration. Nevertheless, in the present study, CC and DC individuals performed below the level of SC individuals. Therefore, a transient phase of congenital visual deprivation does not prevent the use of vision for balance and locomotion later in life, but transient phases of congenital or childhood visual deprivation seem to have long-lasting effects on postural control beyond the time of blindness. These results suggest that vision plays a crucial role in calibrating the proprioceptive and vestibular system.

The differences in balance performance between cataract-reversal individuals compared with sighted controls were more pronounced on hard ground than on soft ground. However, the effect was moderated by visual input. On the one hand, when participants were tested with eyes open, the decrease in performance on soft ground compared with hard ground was more pronounced in CC and DC individuals than in SC individuals. On the other hand, sighted participants showed a large decrease in performance on soft ground in the eyes-closed condition. These results may suggest that SC individuals make more use of visual cues than CC and DC individuals to adjust to the reduced somatosensory feedback on soft ground. When visual input is prevented, SC individuals were no longer able to compensate for the reduced somatosensory input. This idea is further supported by the finding that the better performance of SC participants tested with eyes closed compared with permanently blind individuals (CB and LB) was significant only on hard ground but not on soft ground. However, the ground effects should be interpreted with caution and replicated in further studies, as performance in the eyes-closed condition on soft ground was overall very low in all participant groups. Floor effects may therefore have contributed to a lack of significant group differences in this condition.

### Sensitive period for the development of postural control

The finding that CC participants performed worse in the balance task compared with DC participants provides evidence for the crucial role of vision following birth and thus suggests a sensitive period during the development of postural control. In fact, CC individuals did not differ in balance performance in the eyes-closed condition from CB individuals; thus, both groups performed worse than SC individuals. Sensitive periods are defined as phases during which adequate input is essential for functional (and structural) brain development (Knudsen, 2004). It could be speculated that congenital visual deprivation permanently changes the genuine mechanisms of crossmodal calibration (Bruns et al., 2022). Previous work in visual–vestibular calibration has suggested that the relative weighting of visual and vestibular cues does not depend on cue reliability but rather follows a fixed ratio (Zaidel, Turner, & Angelaki, 2011). This fixed-ratio value seems to emerge under the control of sensory experience (Bruns et al., 2022). Prospective studies in children have revealed that this value is only considered for unsupervised crossmodal recalibration from middle childhood onward (Rohlf, Li, Bruns, & Röder, 2020). Correspondingly, although infants make use of sensory cues to adjust movements and postural control in the first months of life (Dusing, 2016), children under the age of 6 to 7 years were not able to adapt to altered sensory input during balance tasks. These findings suggest that the relative weighting of proprioceptive, vestibular, and visual signals undergoes developmental changes until middle childhood (Forssberg & Nashner, 1982; Foudriat, Difabio, & Anderson, 1993). Here, we speculate that when it comes to crossmodal calibration of postural control in CC individuals, they employ an altered weighting of proprioceptive, vestibular, and visual cues. Their weighting of sensory cues seems to be less efficient than in SC individuals, resulting in worse balance performance in both the eyes-closed condition and the eyes-open condition. Due to the availability of early visual cues in DC individuals, this group's fixed ratio for calibrating postural control might be more similar to that of normally sighted individuals that is; with a higher weight for vision. As a result, DC individuals outperform CC individuals in postural control.

Results on balance performance in children with other congenital ocular anomalies support this reasoning. For example, children with strabismus have been reported to be impaired in postural control (Jayakaran et al., 2021). Some authors have discussed poor balance in patients with strabismus as a consequence of impaired stereovision and reduced visual acuity during sensitive periods when sensory systems are calibrated for postural control (Zipori, Colpa, Wong, Cushing, & Gordon, 2018). However, motor development has been shown to improve rapidly after strabismus surgery (Bucci et al., 2016; Drover, Stager, Morale, Leffler, & Birch, 2008; Legrand et al., 2011), suggesting that the effects of poor stereopsis are more reversible than a total lack of vision. As a note of caution: Studies on the effects of strabismus surgery on motor skills are overall scarce, with highly heterogeneous samples with respect to the patients’ characteristics, including time of surgery and how motor skills were assessed. CC individuals of the present study were carefully selected based on criteria as listed in the Materials and methods section, which makes the presence of visual pattern vision at birth highly unlikely.

If early developmental visual experience is important for the calibration of the vestibular and the proprioceptive systems, one might expect LB individuals to show better balance and gait performance than CB individuals. However, no significant differences between LB and CB individuals were observed in the present study. The small sample size and the considerable heterogeneity in the onset of visual impairment in the late blind group might explain the lack of a significant difference between congenital and late blind individuals.

### Visual acuity and balance performance

Whether impaired vision in CC individuals might explain their reduced balance skills compared with SC individuals warrants discussion. All but one CC individuals had considerable visual impairments (*n* = 8 logMAR visual acuity between 0.5 and 1.3, *n* = 2 logMAR visual acuity worse than 1.3). Furthermore, overall visual acuity was lower in CC participants than in DC participants, and visual acuity was positively correlated with balance performance across all sight-recovery participants. Associations between poor visual acuity and reduced performance in balance tasks have been reported in a large epidemiological study in adults who were 40 years and older (Willis, Vitale, Agrawal, & Ramulu, 2013). These results might point to the importance of reliable visual cues for postural control. However, CC individuals in our study performed worse than SC individuals not only in the eyes-open condition but also in the eyes-closed condition. Moreover, permanently blind participants showed lower balance scores than sighted participants tested with eyes closed. These findings are in line with those of Willis et al. (2013), who reported worse balance performance in participants with reduced visual acuity compared with participants with normal vision in an eyes-closed condition. Thus, reduced visual input during testing did not exclusively explain poor postural control in visually impaired and blind individuals. Similarly, children with strabismus with and without amblyopia were more affected by closing their eyes than normally sighted children during challenging balance tasks, suggesting a visually less well calibrated proprioceptive and vestibular system (Zipori et al., 2018). Willis et al. (2013) speculated that low vision might induce vestibular dysfunctions, probably by altering feedback loops involved in the vestibular–ocular reflexes, which affect balance even when eyes are closed. Taken together, findings of poor balance performance in individuals with low vision of different causes not only in eyes-open conditions but also in eyes-closed conditions suggest that visual impairments cause lasting changes in postural control.

### The role of physical exercise for balance skills

Both CB and LB participants showed reduced balance performance compared with sighted participants tested with eyes closed. Thus, permanently blind participants were not able to fully compensate for the lack of visual calibration of the vestibular and proprioceptive system, even after many years of blindness. A low level of physical activity in individuals with visual impairments might have contributed to this deficit. Blind and visually impaired children and young adults have been reported to engage in less physical activity and to spend more time in sedentary behavior than their peers without visual impairments (Augestad & Jiang, 2015; Houwen et al., 2008; Rogge et al., 2021). In blind children, lower levels of physical activity have been associated with increased postural sway, reduced balance performance, and lower gait stability (Müürsepp et al., 2018; Rogge et al., 2021). However, balance and postural control in blind and visually impaired participants can be improved by training (Rogge et al., 2019; Salar, Ardakani, Lieberman, Beach, & Perreault, 2022). Unfortunately, we did not systematically assess participants’ physical activity. Therefore, future work should examine the role of total past and present physical activity for postural control after permanent and transient blindness.

### Effects of permanent and transient phases of blindness on gait parameters

In contrast to the finding of poor balance performance in sight-recovery and permanently blind participants, visual deprivation was not associated with reduced gait speed and gait stability in the present study. CB and LB participants made even longer strides than SC individuals tested with eyes closed, and LB individuals showed less variability in gait parameters than SC individuals, suggesting that permanently blind participants were able to compensate for the absence of vision while walking. This is partially in contrast to previous studies reporting no differences in gait speed between blind participants and normally sighted participants with eyes closed (Hallemans et al., 2010; Rogge et al., 2021). However, there are some results showing less sway in CB individuals when they are standing on solid surfaces (Schwesig et al., 2011) and faster reaction times to displacements of the underground, which might play a role in detecting obstacles and avoiding hazards while walking without vision (Nakata & Yabe, 2001). The dissociation between lower balance performance and unimpaired walking patterns in sight-recovery and permanently blind participants might be due to daily walking practice. In contrast, standing on one leg, as in the balance task, was rather unfamiliar to most participants. However, one must keep in mind that walking with a blindfold was a highly unfamiliar task for both the normally sighted as well as the sight-recovery individuals, who might have adapted a more cautious walking strategy during the gait assessment. Future studies should increase the number of walking trials to test whether the sight-recovery and normally sighted individuals are capable of adapting to a blindfold.

### Limitations

One limitation of the present study is that we report cross-sectional data rather than repeated (longitudinal) assessments before and after cataract-removal surgery. Future studies should track the time course and mechanisms (e.g., changes in sensory cue weighting) following sight restoration. Finally, the sample size was rather small, which lowers the statistical power of the present study. We had clear a priori hypotheses based on previous findings reporting altered crossmodal calibration after transient visual deprivation. However, considering the small sample size and the lack of studies on balance and gait after transient visual loss, the present study encourages future research with larger sample sizes. Such additional research would allow investigating possible associations between the recovery in balance performance and, for example, the duration of visual deprivation and time since surgery.

## Conclusions

The present study investigated the role of visual experience after birth for postural control and locomotion. Sight-recovery individuals were able to make use of visual information for both balance and gait, regardless of whether they were born blind or acquired visual impairments in childhood. The observation that CC individuals did not reach the balance scores of SC and DC individuals, not even when the balance scores were measured while they wore a blindfold, highlights the role of early visual input in calibrating the proprioceptive and the vestibular systems.

## Supplementary Material

Supplement 1
