# Supplementary material for: Postural control depends on early visual experience
Source: J Vis. 2024 Sep 3;24(9):3. doi: 10.1167/jov.24.9.3 (PMC11373724; doi:10.1167/jov.24.9.3)
Supplement: Supplement 1 [file jovi-24-9-3_s001.pdf]

Supplementary Material - Results of non-parametric tests

Balance Performance: Single Leg Stance

Sight-Recovery Participants and Normally Sighted Controls, Eyes Open and Eyes Closed

(parametric bootstrapping for mixed-effects Anovas)

Group	$\chi^2(2, n = 35) = 38.30, p < 0.001$
Condition	$\chi^2(1, n = 35) = 65.01, p < 0.001$
Vision	$\chi^2(1, n = 35) = 85.86, p < 0.001$
Group x Condition x Vision	$\chi^2(2, n = 35) = 17.35, p < 0.001$

All Participant Groups, Eyes Closed

(parametric bootstrapping for mixed-effects Anovas)

Group	$\chi^2(4, n = 58) = 47.49, p < 0.001$
Condition	$\chi^2(1, n = 58) = 61.16, p < 0.001$
Group x Condition	$\chi^2(4, n = 58) = 50.25, p < 0.001$

Gait parameters

Sight-Recovery Participants and Normally Sighted Controls, Eyes Open and Eyes Closed

(parametric bootstrapping for mixed-effects Anovas)

Stride length

Group	$\chi^2(2, n = 34) = 0.25 , p = 0.899$
Vision	$\chi^2(1, n = 34) = 59.67, p < 0.001$
Group x Vision	$\chi^2(2, n = 34) = 0.73, p = 0.718$

Stride length variability

Group	$\chi^2(2, n = 34) = 0.07, p = 0.966$
Vision	$\chi^2(1, n = 34) = 23.02, p < 0.001$
Group x Vision	$\chi^2(2, n = 34) = 4.76, p = 0.111$

Stride time

Group	$\chi^2(2, n = 34) = 0.11, p = 0.950$
Vision	$\chi^2(1, n = 34) = 34.62, p < 0.001$
Group x Vision	$\chi^2(2, n = 34) = 3.56, p = 0.192$

Stride time variability

Group	$\chi^2(2, n = 34) = 1.5, p = 0.483$
Vision	$\chi^2(1, n = 34) = 16.14, p < 0.001$
Group x Vision	$\chi^2(2, n = 34) = 5.43, p = 0.099$

Gait speed

Group	$\chi^2(2, n = 34) = 0.42, p = 0.828$
Vision	$\chi^2(1, n = 34) = 67.07, p < 0.001$
Group x Vision	$\chi^2(2, n = 34) = 0.99, p = .653$

Gait speed variability

Group	$\chi^2(2, n = 34) = 1.67, p = 0.473$
Vision	$\chi^2(1, n = 34) = 1.61, p = 0.228$
Group x Vision	$\chi^2(2, n = 34) = 8.55, p = 0.026$

Sight-recovery participants did not differ significantly from normally sighted controls in mean minimum toe clearance, variability of minimum toe clearance and local dynamic gait stability (LDE) [main effects of Group and Group x Vision interactions all $\chi^2 < 2.06$, all $p > 0.39$].

All Participant Groups, Eyes Closed

(Kruskal-Wallis Test)

Stride length

Group	$\chi^2(4, n = 54) = 17.18, p = 0.002$

Variability of stride length

Group	$\chi^2(4, n = 54) = 12.53, p = 0.014$

Gait speed

Group	$\chi^2(4, n = 54) = 5.79, p = 0.216$

Variability of gait speed

Group	$\chi^2(4, n = 54) = 14.23, p = 0.007$

Groups did not significantly differ in stride time, minimum toe clearance and local dynamic gait stability (LDE), neither in mean values nor in the variability of these gait parameters [all $\chi^2 < 7.57$, all $p > 0.11$].
